# Predictors of adherence in Austrian employees during the COVID-19 pandemic: results of an online survey

**DOI:** 10.3389/fpubh.2024.1347818

**Published:** 2024-03-01

**Authors:** Alexander Avian, Clemens Könczöl, Bettina Kubicek, Ulrike Spary-Kainz, Andrea Siebenhofer

**Affiliations:** ^1^Institute for Medical Informatics, and Statistics and Documentation, Medical University of Graz, Graz, Austria; ^2^Institute of Psychology, University of Graz, Graz, Austria; ^3^Institute of General Practice and Evidence-based Health Services Research, Medical University of Graz, Graz, Austria; ^4^Institute of General Practice, Goethe University Frankfurt, Frankfurt am Main, Germany

**Keywords:** COVID-19, adherence, health belief model, employees, social norms, corona fatigue, barriers to health-promoting measures, online-survey

## Abstract

**Background:**

Since the beginning of the pandemic in December 2019, Coronavirus disease 2019 (COVID-19) has been a significant challenge to health care systems throughout the world. The introduction of measures to reduce the incidence of infection had a significant impact on the workplace. Overall, companies played a key and adaptive role in coping with the pandemic.

**Methods:**

Cross-sectional data from an online-survey of 1,183 employees conducted during the COVID-19 pandemic in spring 2021 in Austria were used in the analyses. The influence of health beliefs (e.g., perceived severity), modifying factors (e.g., age) and time-dependent factors (e.g., corona fatigue) on individual adherence were evaluated. The conception of the questionnaire was based on the health belief model.

**Results:**

The majority of respondents were female (58.3%), worked in companies with more than 250 employees (56.6%) and had been to an academic secondary school or had a university degree (58.3%). Overall, employees were adherent to most of the measures at their company (>80%), except for wearing FFP-2 masks when they were travelling in a car with coworkers (59.3, 95%CI 51.3–66.7%). Overall adherence was associated with high ratings for the meaningfulness of testing (OR: 2.06 95%CI: 1.00–4.22; *p* = 0.049), the extent to which social norms govern behavior (OR: 6.61 95%CI: 4.66–9.36; *p* < 0.001), lower perceived difficulties associated with the adoption of health-promoting measures (OR: 0.37 95%CI: 0.16–0.82; *p* = 0.015) and lower corona fatigue (OR: 0.23 95%CI: 0.10–0.52; *p* < 0.001). Adherence to four single measures was influenced by different predictors. The most important predictors (important for the adherence to three out of four single measures) were social norms and corona fatigue.

**Conclusion:**

The importance attached to testing and social norms, as well as lower perceived barriers to health-promoting measures and low levels of corona fatigue all increase overall adherence to Covid-19 protective measures in companies. Strategies to improve adherence should be adapted depending on the aim (to raise overall adherence or adherence to individual measures) and on the group of persons that is being targeted.

## 1 Introduction

Since the beginning of the pandemic in December 2019, Coronavirus disease 2019 (COVID-19) has been a significant challenge to health care systems throughout the world (1–9). To slow down transmission rates, almost all governments developed a prevention strategy of some kind, as recommended by the World Health Organization (WHO) (10).

The corona pandemic and associated measures have become a global public health issue and have disrupted people’s daily lives and work, and severely damaged the global economy (11, 12). Companies were faced with such hitherto unknown challenges as the requirement to close down facilities, deal with greater numbers of employees off sick (13), and further health-promoting measures such as short-time work, teleworking (14), and personal protection (e.g., wearing masks, testing, distancing). As early as March 2020, the WHO issued simple recommendations to employers that were aimed at preventing the spread of Covid-19 (15), and other organizations such as the International Labor Organization followed suit with additional guidance for employers on how to protect their employees from infection in the workplace (16, 17).

The introduction of measures to reduce the incidence of infection had a significant impact on the workplace. While the closure of enterprises had a direct economic affect, the wearing of masks, the introduction of tests and the opportunity to work from home at least enabled enterprises to continue operating at reduced risk of infection for employees. A rapid review and meta-analyses published by Ingram provided evidence that a combination of SARS-CoV-2 infection prevention and control measures resulted in fewer Covid-19 infections among employees, especially when timely and widespread contact tracing and isolation was combined with smaller worker cohorts and adequate personal protective equipment (18).

Overall, companies played a key and adaptive role in coping with the pandemic. One longitudinal study of full-time employees in Japan showed that the implementation of health-promoting measures in companies varied over time, with an increase in the first phase in spring 2020 (19), steady rates during the summer, and a decrease in preventive measures between summer and November 2020 (20). We also know that adherence to some measures in the general population steadily decreased towards the end of the pandemic (21–23). Already at the beginning of the pandemic from April to May 2020 adherence decreased from 66%% to 33–38% in a Norwegian cohort (23). In a Spanish cohort, it could be shown that less people showed preventing behavior like disinfecting surfaces (42% vs. 55%) and washing hands often with soap and water (81% vs. 85%) in the end of 2020 compared to 2 months before (22). Adherence data from employees at work are rare, but a huge study of over 50,000 people performed in the UK showed that low rates of teleworking were associated with lower than average compliance, and high rates of teleworking with higher than average compliance with preventive measures (24).

The health belief model (25) offers one way to explain individual health behavior like adherence to measures. This model includes factors such as perceived susceptibility, perceived severity, perceived benefits, perceived barriers and cues to action, has often been used to explain differences in adherence. Zewdie et al. (26) concluded in their review that the health belief model can explain a large part of the variation in people’s behavior regarding COVID-19 preventive behavior with perceived benefit being the most important predictor, followed by self efficacy. Looking on specific behaviors Limbu et al. (27) showed in their review, that COVID-19 vaccine hesitancy reinforced by perceived barriers and mitigated by perceived benefits, perceived severity. Conflicting results were found for perceived susceptibility. Self-efficacy has only rarely been studied and has also shown an mitigated effect. Regarding modifying variables female sex was found to increase vaccine hesitancy. Other modifying factors have rarely been examined (e.g., social norms) or shown conflicting results (income). Liang et al. (28) investigated in their review different behaviors (hand hygiene, face mask wearing, physical distancing) and found that knowledge, positive attitudes and perceived norms increased adherent behavior for all three examined behaviors. Perceived susceptibility showed no effect, perceived severity and perceived control mixed effects. Self-efficacy (increased adherent behavior) and negative attitudes (no effect) were only investigated for hand hygiene and physical distancing. Aside from these population-wide studies, there are less studies that explicitly address the workplace and these mostly relate to the medical sector [e.g., (29–31)] or students [e.g., (32, 33)]. This study is based on the adapted health belief model (34) and aims to confirm known and identify new factors influencing adherence at the workplace among employees in Austria.

## 2 Methods

Cross-sectional data from an online-survey of 1,183 employees conducted during the COVID-19 pandemic in spring 2021 in Austria were used in the analyses. The questionnaire was based on the health belief model (HBM) and adapted for use in COVID-19 research by Hsing et al. (35) and Siebenhofer et al. (34). In the present version, health behavior (e.g., adherent behavior) is explained by health beliefs beliefs (e.g., perceived severity), modifying factors (e.g., age) and time-dependent factors (e.g., corona fatigue). According to national legislation and institutional requirements, no ethical approval was required for this study.

### 2.1 Questionnaire

The final questionnaire consisted of general items to be answered by all participants and items to which responses were only required from those that had given certain answers to the general questions. All employees were asked about their perceived susceptibility (1 item), perceived severity (3 items), corona fatigue (6 items) (36), meaningfulness of measures (7 items), information and participation at their company (2 items) (34), specific measures (8 items), social norms (1 item), and company support (1 item), as well as their individual work situation (e.g., whether an employee worked in the same room as others, dealt directly with customers etc.). Employees from which more detail was required were asked further questions about perceived barriers (12 items), social norms (4 items), company support (2 items), and specific measures (13 items). A translated version of the German questionnaire can be found in the Supplement. Explorative factor analysis (VARIMAX rotation) was carried out separately for all aspects apart from sociodemographic variables and aspects about which only one question was asked. Internal consistency (Cronbach’s alpha) was calculated for each factor.

#### 2.1.1 Adherence

Since the working situation differed between respondents and therefore involved different measures, respondents’ adherence also varied. Employees’ responses varied between 0 measures (9 employees) and 7 (1 employee). Most employees reported 2 (*n* = 434) or 3 (*n* = 433) measures. Since it made little sense to add up these responses to provide an overall score, employees were divided into two groups. The first group consisted of employees that were adherent to all measures that were relevant to them, while employees that did not follow all relevant measures were assigned to the other group (non-adherent).

#### 2.1.2 Health beliefs

Ten items were used to assess three aspects of the adapted health belief model (perceived severity, perceived susceptibility, perceived barriers). To assess perceived severity, respondents were first asked to compare COVID-19 to influenza (response format: harmless / comparable / more dangerous), and then to assess their personal health risk and the economic risk resulting from measures to combat the coronavirus on a 5-point Likert type response scale. No satisfactory result could be achieved in the factor analysis of perceived severity. The three perceived severity items were therefore analyzed separately.

Perceived susceptibility was assessed using a single item (response format: not at all / slightly / high). To evaluate perceived barriers due to health-promoting measures, respondents were asked whether they were required to adhere to specific measures (e.g., testing themselves before coming to work; checking whether customers had a negative corona test; wearing FFP-2 masks when driving with others; wearing FFP-2 masks at work). If a respondent was expected to carry out at least one of these measures, one of them was randomly chosen and he or she was asked whether they thought the measures were annoying, unnecessarily strict, would prevent the virus from spreading, had been scientifically proven to be effective, violated legal regulations, and were feasible in reality (response format: yes / partly / no). The first four items could be assigned to one factor (Cronbach’s α =0.855). The other two items were assigned to another (practicability of health-promoting measures), whereby the latter had too little internal consistency (α = 0.343) to be considered in the further analysis.

#### 2.1.3 Modifying factors

The following demographic variables were assessed: age (years), gender (female, male, other), educational levels (EL1: compulsory education including those with no school leaving certificate, EL2: apprenticeship, EL3: higher vocational education, EL4: academic secondary school, EL5: university) and number of employees at the company [less than 10 (micro companies) – 10 to 49 (small companies) – 50 to 249 (medium-sized companies) – 250 and more (large companies)].

#### 2.1.4 Time-dependent factors

The corona fatigue aspect contained all six items from Lilleholt’s corona fatigue questionnaire (36) and has a two-dimensional structure (information fatigue, behavioral fatigue). Since this survey was part of a bigger project involving an additional telephone survey, the response formats were adapted to make responses to a telephone survey easier. In this study, the response format was simplified to: agree / partly agree / do not agree. Since the factors proposed by Lilleholt et al. (36) only had an internal consistency of α = 0.717 (information fatigue) and α = 0.695 (behavioral fatigue), exploratory factor analysis was also used to analyze the six items. This resulted in a one-factor model (α = 0.811).

### 2.2 Survey

The questionnaire was transferred to SurveyMonkey. Potential respondents came from one Austrian state (Vorarlberg), whereby smaller companies were selected by the Chamber of Commerce (membership mandatory for all companies) and Chamber of Labor (membership mandatory for all employees) and the Vorarlberg Society for General Practice, and larger companies by members of the works council and the staff manager. The cooperating institutions contacted the potential respondents independently. Therefore, on the one hand, there could have been multiple contacts with the same person through different institutions. On the other hand, it may also be that certain groups of people were not contacted. Furthermore we had no control whether or not reminders were send to potential respondents. Media channels (newspapers and television) were also used for promoting the study. Potential respondents answered the survey from June 1 to June 27, 2021. The online-survey was conducted by a professional research center (L&R Sozialforschung).

### 2.3 Statistics

Baseline characteristics (demographic variables) are presented as mean ± SD or median (IQR), as appropriate. Categorical variables are provided as absolute and relative numbers. In order to identify independent predictors for adherent behavior (outcome of interest) respondents were grouped into an “adherent group” and a “non adherent group.” The adherent group consisted of employees that were adherent to all measures that were relevant to them, while employees that did not follow all relevant measures were assigned to the non-adherent group. In a first step, univariate logistic regression analysis was performed, with adherence serving as the outcome (adherent group vs. not adherent group). Predictors were the factors and the single-item aspects described above, along with sociodemographic variables. To enhance comparability, all dichotomous factors and single-item aspects apart from age were transformed to fit into a range of 0 to 1. To ensure the resulting betas were comparable, the age variable was therefore divided by 100. Significant univariate predictors were checked for multicollinearity (variance inflation factor < 2.5). Remaining variables were included in a multivariate regression analysis (backwards selection). Exploratory also the influence of the predictors on each individual measure (logistic regression analysis: Model 1: outcome: testing for work; model 2: outcome: wearing of FFP2 masks; model 3: outcome: treatment of customers; model 4: outcome: social distancing). For this analysis, significant univariate predictors were also checked for multicollinearity (variance inflation factor < 2.5). The remaining variables were subjected to multivariate logistic regression analysis (backwards selection). SPSS 26 was used in data analysis (IBM Corp, 2019), and a value of *p* < 0.05 was considered significant.

## 3 Results

### 3.1 Demographics

Overall 1,183 employees answered the survey. The majority of respondents were female (*n* = 690, 58.3%), worked in companies with more than 250 employees (*n* = 669, 56.6%) and had been to an academic secondary school or had a university degree (*n* = 690, 58.3%). Detailed demographic information is provided in Table 1.

**Table 1:** Baseline characteristics of responders (*n* = 1,183).

	*n* (%)
*Gender*	
Female	690 (58.3%)
Male	463 (39.1%)
Others	6 (0.5%)
Misssing	24 (2.0%)
*Age*	
<30 years	201 (17.0%)
30–39 years	293 (24.8%)
40–49 years	288 (24.3%)
≥50 years	376 (31.8%)
Missing	25 (2.1%)
*Number of employees*	
<10	72 (6.1%)
10–49	145 (12.3%)
50–249	243 (20.5%)
≥250	669 (56.6%)
Missing	54 (4.6%)
*Educational levels*	
EL5: University	373 (31.5%)
EL4: Academic secondary school	317 (26.8%)
EL3: College for higher vocational education	233 (19.7%)
EL2: Apprenticeship	212 (17.9%)
EL1: Compulsory education including those with no school-leaving certificate	13 (1.1%)
Missing	35 (3.0%)
*Division*	
Manufacturing	137 (11.6%)
Trade (wholesale, retail)	46 (3.9%)
Traffic and transport	43 (3.6%)
Services	78 (6.6%)
Public administration, defence and welfare funds	436 (36.9%)
Health and social care	206 (17.4%)
Others	161 (13.6%)
Missing	76 (6.4%)

**Figure 1:**
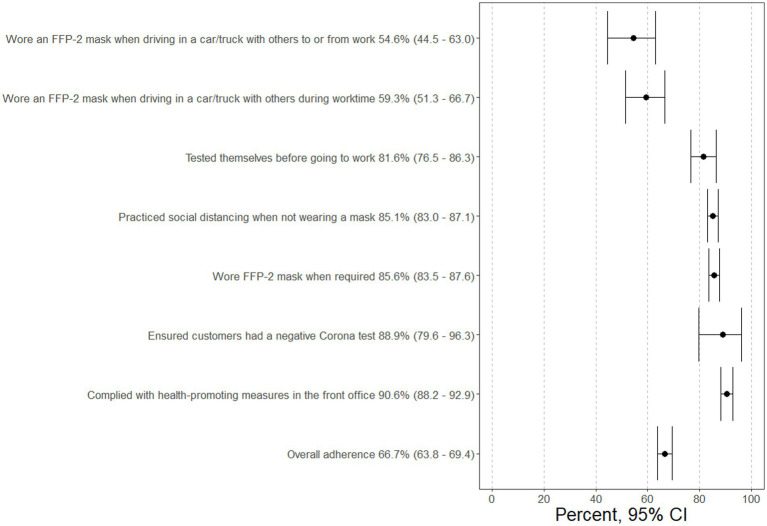
Percentage of people adherent to individual measures.

Overall, employees were adherent to most of the measures at their company, except for wearing FFP-2 masks when they were travelling in a car with coworkers (in a car during worktime: 59.3, 95%CI 51.3–66.7%; in a car on the way to work: 54.6, 95%CI: 44.5–63.0%). The other measures were adhered to by more than 80% of employees (Figure 1). Furthermore 60.4% (95%CI: 57.4–63.2%) said their co-workers adhered to the measures at their company.

The majority of the employees rated a COVID-19 infection as more dangerous than an influenza infection (more dangerous: 69.7%, comparable 25.3%, harmless 1.4%). We also measured personal health risk (9.0% very high, 21.0% high, 45.7% moderate, 16.4% low, 5.2% very low), economic risk stemming from measures to combat the coronavirus (15.0% very high, 25.0% high, 30.7% moderate, 16.8% low, 10.1% very low) and perceived susceptibility (high 15.6%, slight: 69.7%, non-existent: 13.3%). Overall employees rated perceived barriers due to health-promoting measures as low (median 0.25, IQR: 0.10–0.50) and their corona fatigue as moderate (0.50, 0.33–0.67). While testing for the coronavirus was considered important (median 0.83, IQR: 0.67–1.00) wearing FFP-2 masks was rated as moderately important (0.50, 0.17–0.83).

### 3.2 Influence on adherence

In a first step, the following variables were significant univariate predictors of adherence to company-specific health-promoting behaviors: age, gender, and responses to all three perceived severity items (comparison to influenza, personal health risk, economic risk), perceived susceptibility, perceived barriers due to health-promoting measures, social norms, corona fatigue, meaningfulness of wearing FFP-2 masks, meaningfulness of testing, number of employees at the company, company support to help employees follow the measures, and the provision of information by the company (Supplementary Figure S1; Supplementary Table S1).

**Figure 2:**
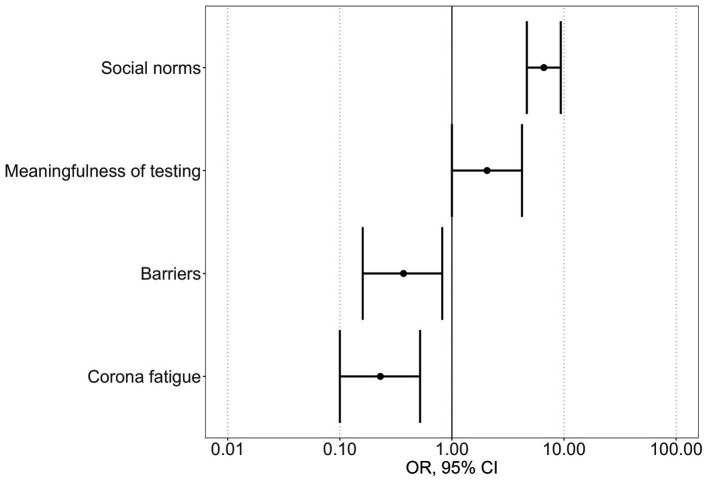
Independent multivariate predictors of adherent behavior.

In a second step, multivariate regression analysis indicated that four independent predictors explained 31% of the variation in adherence (*R*^2^_Nagelkerke_ = 0.308). Increased health-promoting behaviors were also associated with high ratings for the meaningfulness of testing (OR: 2.06 95%CI: 1.00–4.22; *p* = 0.049), the extent to which social norms govern behavior (OR: 6.61 95%CI: 4.66–9.36; *p* < 0.001), lower perceived difficulties associated with the adoption of health-promoting measures (OR: 0.37 95%CI: 0.16–0.82; *p* = 0.015) and lower corona fatigue (OR: 0.23 95%CI: 0.10–0.52; *p* < 0.001) (Figure 2).

### 3.3 Influence on single measures of adherence

Multivariate regression analysis of individual measures indicated that two to four independent predictors explained 9 to 50% of variance. Seven different predictors were included in the final four models. No predictor was included in all the final models. The predictors that were most often included were social norms (three times) and corona fatigue (three times) (Table 2; Supplementary Figure S2).

**Table 2:** Independent multivariate predictors of adherence to various measures (OR and 95%CIs are provided in parentheses).

	Model 1- outcome: testing for work	Model 2- outcome: wearing of FFP2 masks	Model 3- outcome: treatment of customers	Model 4- outcome: social distancing
Age				1.03 (1.02–1.05)
Social norms	3.19 (1.57–6.45)	7.91 (4.79–13.07)	3.55 (1.81–6.95)	
Barriers				0.28 (0.13–0.63)
Corona fatigue	0.22 (0.06–0.82)	3.39 (1.24–9.29)		0.26 (0.10–0.68)
Meaningfullness wearing FFP-2 masks		10.58 (4.93–22.69)		
Company support		38.92 (21.76–69.59)		
Meaningfullness testing			3.33 (1.26–8.80)	

## 4 Discussion

This cross-sectional online survey of 1,183 people working in Vorarlberg, Austria, during the COVID-19 pandemic in spring 2021 showed that social norms and corona fatigue were independent predictors of greater adherence in general and adherence to most of the investigated behaviors in particular. Further independent predictors of general adherence were higher perceived meaningfulness of testing and lower perceived barriers due to health-promoting measures. Depending on which Covid-19 protective measure in companies is considered, additional factors such as age, meaningfulness for wearing FFP2-masks and company support may also be relevant for individual measures.

### 4.1 Social norms

In our study, social norms – the informal rules that govern behavior in groups and societies and hence concern that individuals or groups may disapprove of one’s conduct (37) – were strong predictors of generally adherent behavior during the COVID-19 pandemic. They specifically influenced testing behavior, mask wearing and treatment of customers, but had no impact on social distancing in the work environment. These results generally agree with various other studies in the health sector [e.g., McEachan et al. (38)], as well as with studies on COVID-19 in particular [e.g., Shanka and Gebremariam Kotecho (39)]. Studies on adherence to COVID-19 measures have shown that adherence decreases when others are seen not wearing a mask and when people are heard speaking disparagingly about wearing one (40). Furthermore it has been reported that social norms influence social distancing (41), hand washing (42) and both vaccine hesitancy (43) and actual vaccinations (44) in the general population. Furthermore, professional behaviors such as the use of personal protective equipment is influenced by social norms (45).

With regard to prescribed COVID-19 measures, reactance phenomena may have encouraged the formation of social norms in specific groups (40). Reactance refers to an unintended behavioral reaction to a stimulus that occurs because an individual’s sense of behavioral freedom is threatened (e.g., decrease in adherence after the introduction of new measures). Since reactance to COVID-19 measures is associated with political discontent (46), groups that are opposed to the political authorities in their country and the measures they introduce tend to show reactance, thus reinforcing their own social norms. As concluded by Resnicow et al. (47), a subgroup’s general propensity to defy authority or oppose any laws or public health advice on the basis that it impinges upon personal rights will automatically lead to a rejection of, for example, COVID-19 protective practices. Reactance is therefore a reflection of the social norms in this subgroup of the population.

An important example of how social norms can affect adherence is the way in which social norms predict individuals’ information avoidance (48). Information avoidance is influenced by subjective norms (what people important to me think I should do), descriptive norms on a personal level (what people important to me are doing), injunctive norms on a personal level (what people important to me think should be done) and injunctive norms on a societal level (what the majority of people think should be done) but not descriptive norms on a societal level (what the majority of people actually do) (49). In addition to the norm itself, the origin of information about the behavior of relevant groups and their expectations, as well as information about the pandemic in general, also plays a role. In a meta-analysis, Li (50) showed that channel belief (perceived trustworthiness and usefulness of information) was most responsible for explaining information avoidance. Depending on the groups that are relevant to an individual, specific sources of information are trusted and therefore sought out, while others are rejected, or discussion partners are avoided or preferred. Furthermore, it can be observed that when information is sought in a targeted manner (intentional exposure), e.g., using a trusted channel, people are more likely to believe it than when they come across it by accident (incidental exposure) (51).

Interestingly, the effect of social norms can change over time. Zhou et al. (52) showed that social norms had more influence on individual behavior during the first and second waves of COVID-19 than on later waves. In their study, social norms were defined as the assumed behavior of neighbors, which would seem to imply that the reference group (neighbors) was more important at the beginning of the pandemic than later, when other groups may have established themselves as references. Borkowska and Laurence (53) had similar results and were able to show that for certain groups (certain ethnic minority groups, lower-skilled) perceived cohesion in the neighborhood decreased during the pandemic, apparently indicating that the neighborhood had lost importance as a reference group for social norms.

### 4.2 Corona fatigue

Another important factor determining the level of adherence in a population is so-called corona fatigue, which was associated with decreased adherence to health-promoting measures in our study. According to the World Health Organization (WHO) definition, coronavirus fatigue is the emotional exhaustion caused by feeling distressed or frustrated by the pandemic and “sustained and unresolved adversity” (54). Such emotional exhaustion corresponds more or less with items assessing behavioral fatigue. Studies have shown that adherence is negatively influenced by information fatigue (55, 56), behavioral fatigue (34) and pandemic fatigue in general (57).

In addition to the influence of corona fatigue on adherence behavior, corona fatigue also appears to increase over time. A sharp increase in corona fatigue (58) was particularly marked following the launch of the vaccination program. However, it may be short-sighted to regard this particular increase as a sign of fatigue, as, for example, there are indications that interest in finding out more about the pandemic did not decrease overall, but the focus shifted from scientific to political topics (59). Another aspect is also the coverage of this topic within medias. The topic of corona fatigue is therefore accompanied by the so-called “issue fatigue.” This means that a topic that is very prominent in the media is replaced after a while by other, more important topics. As the media competes for attention, they adapt to the mood and report less or differently on topics in which society has lost interest. Changes during the pandemic have an impact on the attention the pandemic receives from media and therefore issue fatigue contribute to the pandemic’s decreased newsworthiness (60). The interaction between issue fatigue and corona fatigue is described very well in a study of young adults (61). While the beginning of the pandemic was characterized by frequent press conferences and a daily update of COVID-19 cases, which was experienced as a phase of shock by young adults, corona fatigue later emerged. In this phase, the information was dosed. Due to emotional and informational overload, the young adults tried to regain control of their lives by determining how much information they consumed. Over time, the media began to focus more on other topics and young adults began to consume more news. This phase was referred to as “back to normal.” In addition to the effect that less news is consumed due to corona fatigue, the risk of misinformation acceptance also increases (62).

It is also important to mention that different groups were not affected by pandemic fatigue to the same extent. In the literature, female gender, younger age, economic inactivity, a low level of education, lower resilience and poorer coping abilities, greater fear/anxiety in connection with COVID-19, and poorer health were found to increase pandemic fatigue (63–69). For one group, the changes in their work and especially in the appreciation of their work were particularly dramatic. While at the beginning of the pandemic the work of medical staff, and especially nurses, was viewed as very valuable, this appreciation was often not reflected in their salaries. In this group, the corona fatigue was accompanied by exhaustion, disappointment and sometimes defeatist behavior (70).

### 4.3 Strengths and limitations

Although some studies have evaluated psychological factors in companies during the pandemic, our study is different in that it evaluates adherence in companies on the basis of the health belief model (25). One study performed in German companies showed that as compared to men, female employees were particularly exhausted during the pandemic (71). Another study conducted in China and based on interviews with 700 employees revealed that employees in the 30–40 age group were particularly exhausted, as were employees with a higher level of education and those with a relatively low family income (72). A longitudinal study of 419 workers in the USA demonstrated that economic vulnerability did not affect adherence to Covid-19 infection protection measures recommended in guidelines. However, cognitive attitudes were strong predictors of compliance with protective measures in the most economically secure class, while worry was a significantly stronger predictor of compliance in the most vulnerable group (73).

Our study has several limitations. The questionnaire was broadly distributed by the office of the Vorarlberg Provincial Government, the Chambers of Commerce and Labor and further groups we contacted such as work councils, personnel managers, safety experts and the Society of General Practice. Nonetheless, selection bias cannot be ruled out. Furthermore, as this was an online survey and only persons willing to participate answered our questionnaire voluntarily, the generalizability of our findings to employees as a whole may be limited. Another limitation is that it cannot be ruled out that other aspects (e.g., trust in state authorities) that could have an influence on behavior were not surveyed and therefore their influence was not examined.

## 5 Conclusion

The results of this Austrian cross-sectional online survey of employees show that combining the health belief model with aspects that vary over time provides useful information on why adherence varies among company employees. The importance attached to testing and social norms, as well as lower perceived barriers to health-promoting measures and low levels of corona fatigue all increase overall adherence to Covid-19 protective measures in companies.

Adherence to individual measures was also influenced by age, importance attached to wearing FFP2-masks and company support, showing that strategies need to vary depending on the particular behavior that is being targeted.

Strategies to improve adherence should be adapted depending on the aim (to raise overall adherence or adherence to individual measures) and on the group of persons that is being targeted (e.g., employees in large or small companies, in manufacturing, the wholesale or retail trade, or in the health sector). Furthermore, the government, employers and, for example, work councils and union representatives can also play an important role in increasing adherence.

## Data availability statement

The raw data supporting the conclusions of this article will be made available by the authors, without undue reservation.

## Ethics statement

The requirement of ethical approval was waived by Vorarlberg Ethics Committee (Ethikkommission Vorarlberg) for the studies involving humans because according to Austrian law, a vote from the ethics committee is not required for surveys that do not specifically include patients. The studies were conducted in accordance with the local legislation and institutional requirements. The ethics committee/institutional review board also waived the requirement of written informed consent for participation from the participants or the participants’ legal guardians/next of kin because since this is an online survey, it can be assumed that only people who want to answer the questionnaire.

## Author contributions

AA: Conceptualization, Data curation, Formal analysis, Methodology, Visualization, Writing – original draft, Writing – review & editing. CK: Writing – original draft, Writing – review & editing. BK: Supervision, Writing – review & editing. US-K: Writing – review & editing. AS: Conceptualization, Funding acquisition, Project administration, Supervision, Writing – original draft, Writing – review & editing.
