# Supplementary material for: Predictors of adherence in Austrian employees during the COVID-19 pandemic: results of an online survey
Source: Front Public Health. 2024 Mar 1;12:1347818. doi: 10.3389/fpubh.2024.1347818 (PMC10940368; doi:10.3389/fpubh.2024.1347818)

Supplemental

**Predictors of adherence in Austrian employees during the COVID-19 pandemic: Results of an online survey**

**Alexander Avian^1^*^,^ Clemens Könczöl^2^, Bettina Kubicek^2^, Ulrike Spary-Kainz**^3^**, Andrea Siebenhofer^3,4^,**

^1^ Institute for Medical Informatics, and Statistics and Documentation, Medical University of Graz, Austria

^2^ Institute of Psychology, University of Graz, Austria

^3^ Institute of General Practice and Evidence-based Health Services Research, Medical University of Graz, Austria

^4^ Institute of General Practice, Goethe University Frankfurt, Germany

*** Correspondence:**alexander.avian@medunigraz.at (AA)

**Supplemental Table 1.** Univariate Results for Overall Adherence

|  | p-value | OR (95%CI) |
| --- | --- | --- |
| Comparison to influenza | <.001 |  |
| comparable | Ref, |  |
| harmless | .369 | 0.628 (0.228-1.732) |
| more dangerous | <.001 | 1.953 (1.483-2.573) |
| Personal health risk | <.001 |  |
| very low | Ref |  |
| low | .006 | 1.929 (1.212-3.070) |
| medium | <.001 | 2.769 (1.803-4.255) |
| high | <.001 | 3.297 (1.998-5.439) |
| Very high | <.001 | 4.248 (2.087-8.649) |
| Economic risk | .008 |  |
| very low | Ref |  |
| low | .294 | 1.244 (0.827-1.870) |
| medium | .632 | 0.910 (0.620-1.337) |
| large | .272 | 0.786 (0.512-1.207) |
| very large | .018 | 0.559 (0.345-0.905) |
| Perceived susceptibility | .002 |  |
| none | Ref |  |
| slight | <.001 | 1.877 (1.324-2.663) |
| high | .006 | 1.858 (1.192-2.897) |
| Meaningfulness of testing | <.001 | 5.644 (3.737-8.526) |
| Meaningfulness FFP-2 masks | <.001 | 5.523 (3.847-7.930) |
| Company support |  |  |
| no support | Ref |  |
| support | .001 | 1.743 (1.243-2.444) |
| Social norms |  |  |
| others do not adhere to protective measures | Ref |  |
| others adhere to protective measures | <.001 | 8.360 (6.334-11.033) |
| Barriers | <.001 | 0.097 (0.059-0.159) |
| Corona fatigue | <.001 | 0.077 (0.046-0.130) |
| Information and participation | <.001 | 3.010 (1.799-5.036) |
| Age/100 | <.001 | 7.610 (2.552-22.694) |
| Female |  |  |
| male | Ref |  |
| female | .001 | 1.524 (1.188-1.955) |
| Number of employees | .004 |  |
| < 10 | Ref |  |
| 10 - 49 | .967 | 1.012 (0.569-1.801) |
| 50 - 249 | .246 | 1.376 (0.802-2.362) |
| ≥ 250 | .022 | 1.797 (1.090-2.692) |
| Educational levels | 0.072 |  |
| EL1/EL2: apprenticeship / compulsory education including  those with no school-leaving certificate | Ref |  |
| EL3: college for higher vocational education | 0.073 | 1.437 (0.967-2.133) |
| EL4: academic secondary school | 0.771 | 0.949 (0.665-1.353) |
| EL5: university | 0.162 | 1.284 (0.904-1.824) |

**Supplemental Figure 1.** Univariate Results for Overall Adherence.

Reference categories: "comparable" in comparison to influenza, "very low" in personal health risk and economic risk, "none" in perceived susceptibility, "no support" in support by company, "others do not adhere to social norms", "male" for gender, "<10" for number of employees and "EL1/EL2" for educational levels


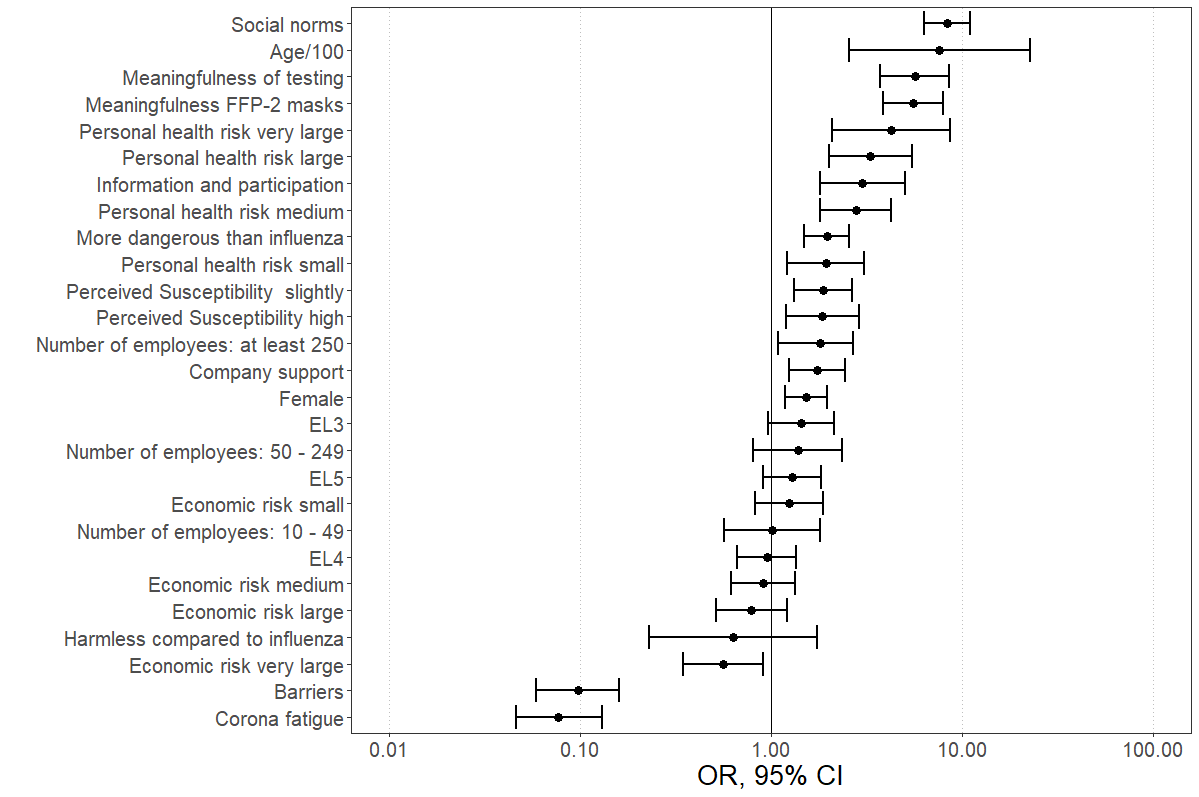


**Supplemental Figure 2.** Multivariate results for various measures


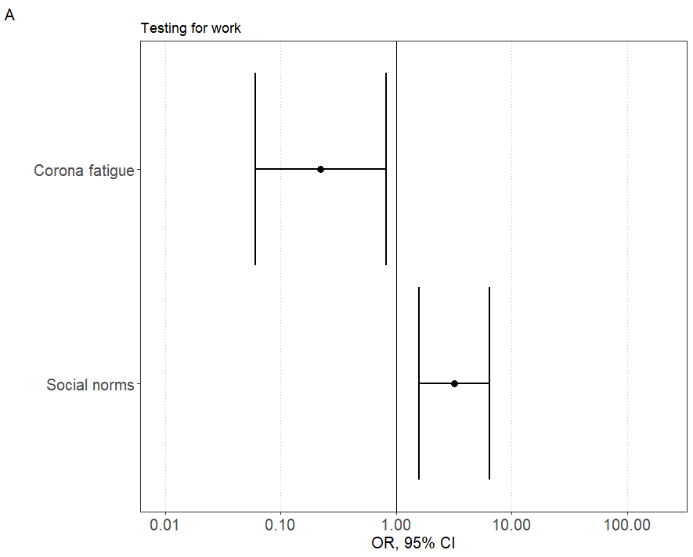

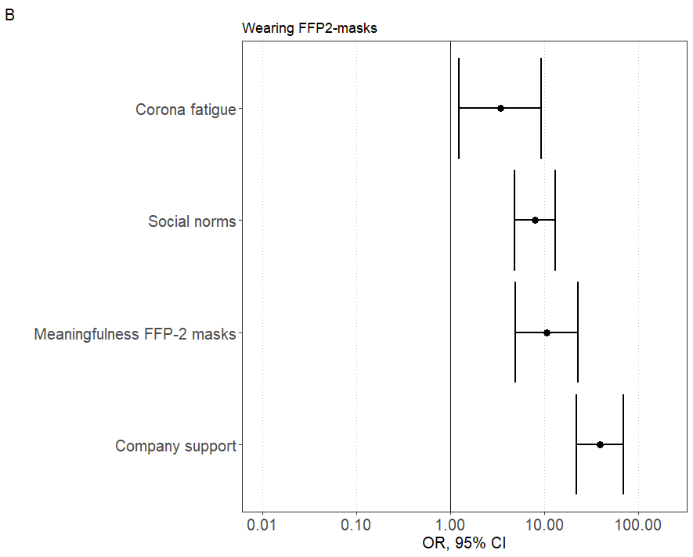

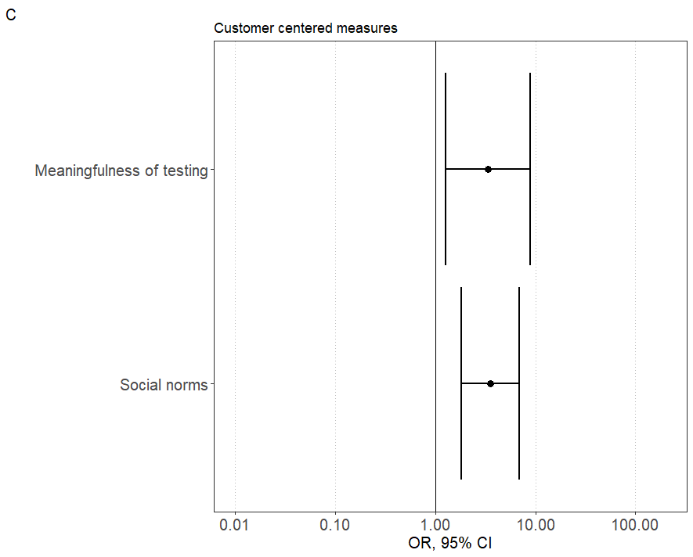

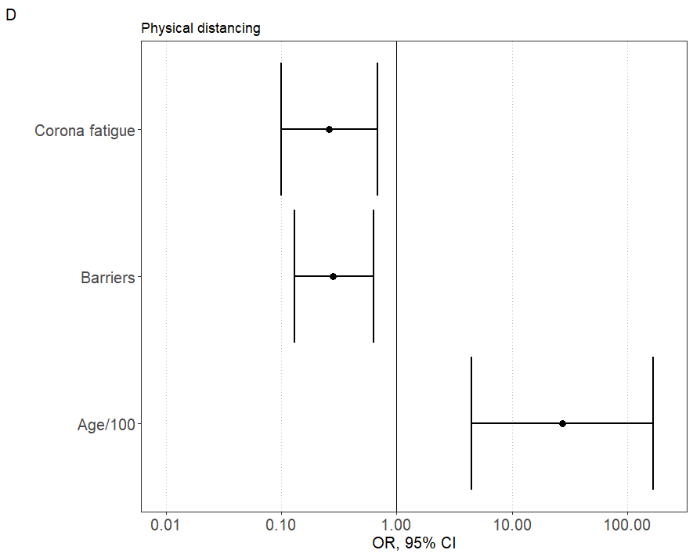

Supplement: Supplementary file 1 [file Table_1.DOCX]
